# Pharmacogenetics testing for poor response to antidepressants: a transnosographic case series

**DOI:** 10.3389/fphar.2024.1440523

**Published:** 2024-10-09

**Authors:** Marie-Agnès Lorvellec, Gilles Sipahimalani, Bertrand Lahutte, Hervé Delacour, Antoine Baldacci, Emeric Saguin

**Affiliations:** ^1^ Department of Psychiatry, Bégin National Military Teaching Hospital, Saint-Mandé, France; ^2^ Ecole du Val-de-Grâce, French Military Medical Academy, Paris, France; ^3^ Biological Unit, Bégin Military Teaching Hospital, Saint-Mandé, France; ^4^ UMR 7330 VIFASOM, Paris, France

**Keywords:** antidepressant, adverse drug reaction, drug response, CYP2D6, CYP2C19, psychiatry, clinical utility, pharmacogenetics

## Abstract

**Introduction:**

Pharmacogenetics (PGx) holds promise for optimizing psychotropic medication use, with *CYP2D6* and *CYP2C19* identified as key genes in antidepressant treatment. However, few studies have explored the genetic variants of these genes in real-world settings for patients experiencing ineffectiveness or adverse drug reactions (ADRs) to antidepressants.

**Methods:**

This case series includes 40 patients who underwent PGx testing due to antidepressant ineffectiveness or ADRs between June 2020 and April 2022. We describe the patients' demographic, clinical, and genetic characteristics and assess the value of PGx testing based on feedback from their psychiatrists.

**Results:**

The most common diagnoses were major depressive disorder (60.0%) and post-traumatic stress disorder (30.0%). Ineffectiveness was reported in 65.0% of patients, ADRs in 2.5%, and both in 32.5%. The antidepressants involved included SSRIs (45.0%), SNRIs (27.5%), atypical antidepressants (20.0%), and tricyclics (17.5%). Only 17.5% of patients had normal *CYP2D6* and *CYP2C19* metabolic activity. Actionable genetic variants were identified in 22.0% of *CYP2D6*/*CYP2C19*-antidepressant-response pairs. PGx recommendations were followed in 92.7% of cases, with significant improvement in ADRs reported in 71.4% of patients and efficacy improvement in 79.5%.

**Discussion:**

Our findings suggest that PGx testing can guide prescribing decisions for patients with antidepressant ineffectiveness or ADRs. The relatively high prevalence of genetic variants affecting pharmacokinetics supports the broader adoption of PGx testing in psychiatric practice.

## 1 Introduction

According to the World Health Organization (WHO), 25% of the world’s population suffers from depression or anxiety every year (World Health Organization, 2017) with these prevalences reaching 31.9% for anxiety and 33.7% for depression during the COVID-19 pandemic (Bello et al., 2022). The economic burden of these pathologies is substantial, hence the need to optimize care to address this growing public health crisis (Chodavadia et al., 2023; de Oliveira Costa et al., 2023).

Antidepressants are the first-line drug treatment for anxiety disorder, major depressive disorder (MDD) and post-traumatic stress disorder (PTSD), in line with scientific recommendations (Sheffler et al., 2023). However, antidepressants are only partially effective. For example, in MDD, the remission rate is only 33% after initial treatment, and 67% after 4 lines of treatment (i.e., the number of different molecules used) (Rush et al., 2006). Moreover, a substantial portion of individuals either do not respond to these medications or encounter adverse drug reactions (ADRs): dizziness, nausea, cardiotoxicity, anticholinergic effects, sexual dysfunction, fatigue and sometimes weight gain (Baldwin et al., 2011). It has been estimated that ADRs occur in over 25% of patients taking antidepressants (Mishra et al., 2013). Consequently, difficulties inherent in prescribing antidepressants often result in longer duration of symptoms, more side effects and increased healthcare costs (Mrazek et al., 2014).

The variability in antidepressant response is influenced by a multitude of factors, with genetic predisposition accounting for approximately 42% of this variability (Swen et al., 2011). The development of pharmacogenetics (PGx) has made it possible to move from non-specific prescribing to personalized prescribing, i.e., choosing a molecule according to the genetic variants identified in the patient. In the case of antidepressants, it has now been established that polymorphisms in the cytochromes P450 2D6 and 2C19 (CYP2D6 and CYP2C19) influence the efficacy and safety of antidepressants (Bousman et al., 2019). Among the over 50 CYP genes involved in drug metabolism, *CYP2C19* (38%), *CYP2D6* (85%), and *CYP3A4* (38%) are most critical for antidepressant metabolism, though their influence can vary with age, sex, and ethnicity (Cacabelos and Torrellas, 2015). Both CYP2D6 and CYP2C19 are highly polymorphic genes which have extensive interactions with the metabolic processes of tricyclic antidepressants (TCAs), selective serotonin reuptake inhibitors (SSRIs), and serotonin-noradrenaline reuptake inhibitors (SNRIs) (Roberts et al., 2023). Two pharmacogenetics companies, Clinical Pharmacogenetics Implementation Consortium (CPIC) and Dutch Pharmacogenetics Working Group (DPWG), have issued prescribing recommendations to adapt doses and molecules to different genetic profiles (Bousman et al., 2023). Based on these recommendations, we recently published our proposal of an algorithm to optimize the choice and dosage of antidepressants using an individual’s genetic profile (Baldacci et al., 2022; Baldacci et al., 2023).

CYP2D6 and CYP2C19 phenotypes vary greatly across the general population. PGx testing allows an individual’s drug metabolic phenotype to be classified into four primary subtypes for CYP2D6: poor metabolizers (PMs), intermediate metabolizers (IMs), normal metabolizers (NMs), or ultrarapid metabolizers (UMs), with a fifth subtype for CYP2C19, as there are also rapid metabolizers (RMs) (Bousman et al., 2023). This valuable information enables clinicians to make personalized decisions regarding drug prescription and dosing for each patient, using their specific genetic data rather than relying solely on average population statistics. Patients who have a RM phenotype may eliminate the treatment too quickly and never reach the therapeutic dose (Jukić et al., 2018; Bousman et al., 2023; Oslin et al., 2022). In contrast, individuals classified as PMs, characterized by reduced enzymatic activity, tend to exhibit higher residual concentrations of antidepressants in serum in comparison to NMs, thereby increasing the likelihood of experiencing ADRs (Schenk et al., 2010; Huezo-Diaz et al., 2012; Chang et al., 2014; Milosavljevic et al., 2021; Jukic et al., 2018; Han et al., 2018).

While the aforementioned studies propose that PGx-guided treatment holds promise in individualizing drug therapies for patients with mental health disorders, many uncertainties are acknowledged in the literature as well. Solomon et al. (2019) conducted a systematic review to explore the potential of PGx testing of CYP2D6 and CYP2C19 to predict antidepressant response. Their findings presented a mix of results, indicating that while PGx testing might predict antidepressant responses in specific individuals, its generalizability to a wider population remains unclear. Solomon et al. (2019) also noted that the lack of positive associations between PGx-guided treatment and antidepressant responses could stem from factors such as studies being underpowered and/or lack of ethnic diversity in the study samples.

A New Zealand team described a case series of 22 individuals who underwent genotyping for severe ADRs to psychiatric medications (Maggo et al., 2019). Authors noted a relatively high but statistically non-significant occurrence of pharmacogenetic variants in patients with ADRs compared to allele frequency surveys conducted in unselected population samples. In another recent case series study of antidepressant responses, variants in 52 individuals, who were prescribed antidepressant treatment and experienced ADRs or ineffectiveness, were described (Kee et al., 2023). They observed that there were only 15% of cases with CYP2D6 and CYP2C19 NM phenotypes. This result is comparable (∼13%) to another study by Hahn and Roll (2023) who carried out a retrospective pharmacogenetic analysis in 108 European adult depressive patients.

Currently, there are very few studies investigating the genetic variants of CYP2D6 and CYP2C19 in a population of patients experiencing either ineffectiveness or ADRs to antidepressants in real-world settings, i.e., longitudinally regardless of the indication of treatment (depression, anxiety disorders, and PTSD). This was therefore the motivation that led us to retrospectively investigate the demographic, clinical and genetic features of patients who underwent PGx testing within our psychiatry department due to ADRs or ineffectiveness to antidepressant treatment between June 2020 and April 2022. Additionally, we aimed to assess the PGx-based prescription recommendations by consulting the treating psychiatrists.

Our hypotheses are outlined as follows: Firstly, we propose that PGx may partially account for ADRs and/or treatment ineffectiveness observed in the population receiving antidepressants. Secondly, we hypothesize that adherence to the prescribing guidelines would be beneficial for clinicians in optimizing patient care. Lastly, we anticipate a high proportion of non-normal metabolizer (non-NM) patients for CYP2D6 and CYP2C19 similar to other observations made previously in this kind of population, considering the history of either ineffectiveness or ADRs to antidepressants.

## 2 Materials and method

### 2.1 Participants

In standard clinical practice in our Psychiatry department, when psychiatrists observe ADRs or ineffectiveness of ongoing antidepressant therapy, they may prescribe PGx testing. Subsequently, the results of this analysis are transmitted to the psychiatrist in the form of a summary detailing CYP2D6 and CYP2C19 phenotypes along with antidepressant prescription guidance accurately predicting dose and improving treatment outcomes (Baldacci et al., 2023). The prescribing psychiatrist may then incorporate this pharmacogenetic guidance into the process of treatment adjustment. However, adherence to these recommendations is not mandatory, allowing for clinical discretion in the optimization of therapeutic strategies tailored to the individual patient’s needs.

In this study, the inclusion criteria were as follows: i) Having undergone PGx testing treatment between June 2020 and April 2022 due to antidepressant ineffectiveness or ADRs, based on the retrieval of all requests from the hospital’s computerized system. ii) Providing informed consent for participation in the study. iii) Receiving a primary diagnosis of MDD, generalized anxiety disorder (GAD), or PTSD. The exclusion criteria covered minors, individuals who declined to participate in the study, and patients who were either receiving their first prescription of antidepressants or had been on treatment for less than 6 weeks.

### 2.2 Genetic analysis

Genetic testing was conducted using peripheral venous blood samples collected in EDTA tubes, in compliance with current legislation and following the acquisition of informed consent from the patient.

For *CYP2D6*, genomic DNA was extracted from peripheral blood leukocytes with the use of the ELITe InGenius^®^ SP 1000 DNA kit (ELITechGroup, Italy) following the manufacturer’s instructions. DNA was eluted in 500 µL of water in the final step and stored at −20°C until required. For *CYP2C19*, PGx testing is performed on EDTA whole blood without prior DNA extraction.

• *CYP2D6* genotyping requires the implementation of two complementary techniques.
  -The first is a strip hybridization technique (PGX-*CYP2D6* Strip Assay kit, Vienna Lab Diagnostics GmbH, Vienna, Austria) for the detection of 22 *CYP2D6* alleles (*CYP2D6* *1 to *12, *14, *15, *17, *29, *35, *39, *640, *41, *58, *114).
  -The second is a real-time PCR technique (*CYP2D6* RealFast CNV Assay kit, Vienna Lab Diagnostics GmbH, Vienna, Austria) for the detection of a deletion (*CYP2D6* *5) or duplication (*CYP2D6* *xN) of *CYP2D6.*

The combined use of these two kits makes it possible to detect 90%–99% of *CYP2D6* alleles observed depending on the ethno-geographic origin of the patients. Among the undetected alleles, only three (*CYP2D6* *36, *49, and *69) are associated with decreased CYP2D6 activity and have an allelic frequency of approximately 1% in the Southeast Asian population (Whirl-Carrillo et al., 2012; PharmGKB, 2017).

• *CYP2C19* genotyping relies on a loop-mediated isothermal amplification technique (LC-*CYP2C19*-LP kit, LaCAR MDX, Seraing, Belgium).

This kit allows targeted detection of the two most common *CYP2C19* alleles (*CYP2C19* *2, *3, and *17). Their absence suggests the presence of the reference allele *CYP2C19* *1, in view of which *1 allele was assigned when no variations were observed. This approach achieves a detection rate of over 93% of *CYP2C19* alleles except in patients of Sub-Saharan African origin (detection rate estimated at 88%). For these patients, targeted detection of the *CYP2C19* *35 allele (associated with null activity) and *CYP2C19* *9 allele (associated with decreased activity) will be requested on a case-by-case basis, increasing the detection rate to over 94% (Whirl-Carrillo et al., 2012; PharmGKB, 2015).

A final verification step involves thorough review by a biologist, who compares the genotyping results translated into phenotyping. To determine phenotypes, it is essential to assess the activity values of both alleles for each pharmacogene (*CYP2D6* and *CYP2C19*). These activity values are available on the PharmGKB website (PharmGKB, 2015; PharmGKB, 2017). The activity score is calculated by summing the values of each allele, allowing for the assignment of a phenotype to individuals, and is presented to psychiatrists in an automatically generated recommendations report (Baldacci et al., 2022; Baldacci et al., 2023).

### 2.3 Data analysis

In our study, we analyzed psychiatrists’ requests for PGx testing from medical records, encompassing patients’ socio-demographic data, the rationale behind genetic testing requests, current diagnoses, and prescribed medications. The results of the PGx tests were obtained from the hospital’s genetics laboratory. Geographical population groups were annotated in accordance with Huddart et al. (2019), distinguishing between several groups including East Asians, Europeans, Oceanians and Sub-Saharian Africans.

Furthermore, we examined prescriptions for potential drug interactions involving cytochrome P450 inducers or inhibitors. We used a list available at https://drug-interactions.medicine.iu.edu/MainTable.aspx (Flockhart et al., 2021) to characterize coprescriptions: Molecules causing a greater than 2-fold increase in plasma AUC (area under the curve) values or a more than 50% decrease in clearance (i.e., moderate and strong inhibitors) were considered CYP2D6 or CYP2C19 inhibitors. Conversely, molecules responsible for a reduction in drug exposure of more than 50% (i.e., moderate and strong inducers) were classified as CYP2D6 or CYP2C19 inducers.

In the subsequent phase, we conducted discussions with the psychiatrists who prescribed PGx tests within our Psychiatry Department, administering a concise questionnaire to gauge the perceived impact of genetic insights on patient management. This questionnaire addressed considerations such as potential modifications in treatment plans and the psychiatrists’ observations on patients’ clinical progress, emphasizing the trade-off between therapeutic efficacy and ADRs. The evaluation of improvement in treatment efficacy and ADRs was facilitated through two visual analog scales, ranging from 0 to 5.

### 2.4 Case analysis

In our case analysis, we adopted the methodology outlined by Hahn and Roll (2023) and Kee et al. (2023) to identify and evaluate gene-drug/antidepressant-response pairs for their clinical actionability. This methodology is described briefly below.

Gene-drug/antidepressant-response pairs were determined by identifying instances where any ineffectiveness or ADRs were reported in relation to each antidepressant. We referred to the CPIC (2023) guidelines, which assign evidence levels A, A/B, or B to antidepressants based on their interaction with the *CYP2D6* or *CYP2C19* genes (Bousman et al., 2023). Utilizing this classification, we extracted gene-antidepressant-response pairs for further analysis, taking into account various types of negative responses. For instance, a case documenting both ADRs and ineffectiveness would contribute two distinct observation pairs within the “CYP2D6/CYP2C19-antidepressant-response” analysis.

Once identified, these pairs were then classified as either “actionable” or “non-actionable,” considering several criteria: the *CYP2D6* and *CYP2C19* pharmacogenes, the specific antidepressants involved, the CPIC evidence levels, and the drug response phenotypes. The criterion of actionability required a critical examination of both clinical and genetic data to ascertain the relationship between genotype-inferred phenotypes, drug exposure, and observed drug response events. Pairs showing a definitive link across these elements were considered “actionable”.

### 2.5 Ethics

The research involving human participants was subjected to review and received approval from the ethics committee (approval number: 2022HJ24). Written informed consent was obtained from all participants prior to their inclusion in the study.

### 2.6 Statistical analysis

We performed descriptive analyses using the Jamovi software (Version 2.4) (The Jamovi Project, 2023). Categorical data are presented as absolute and percentage values. Quantitative data are presented as mean ± SD.

## 3 Results

### 3.1 Descriptive analysis of the population

Our study included 40 patients (26 males and 14 females) with a mean age of 47.7 years. The majority of these patients (n = 33, 82.5%) were of European descent. The primary diagnosis among participants was MDD in 24 patients (60.0%), followed by PTSD in 12 patients (30.0%), GAD in 3 patients (7.5%), and a depressive episode within bipolar disorder in 1 patient (2.5%). Active smoking was reported in 15 patients, representing 37.5% of the cohort.

Treatment outcomes indicated that for 26 patients (65.0%), treatments were deemed ineffective and without ADRs, 1 patient (2.5%) experienced ADRs without ineffectiveness, and 13 patients (32.5%) encountered both ADRs and treatment ineffectiveness, with reported effects including weight gain, libido disorders, sweating and dizziness, among others. These findings are presented in Table 1.

**TABLE 1:** Description of patient sample, PGx testing request reason, and prescribed antidepressant treatment.

	N = 40
Sex	
Male	26 (65.0%)
Female	14 (35.0%)
Age	47.7 (SD 12.4)
Ethnicity	
European	33 (82.5%)
African	3 (7.5%)
Polynesian	2 (5.0%)
Asian	1 (2.5%)
Eurasian	1 (2.5%)
Primary diagnosis	
MDD	24 (60.0%)
PTSD	12 (30.0%)
GAD	3 (7.5%)
Bipolar disorder	1 (2.5%)
Motif of PGx testing	
Cases associated with adverse reactions only	1 (2.5%)
Cases associated with ineffectiveness only	26 (65.0%)
Cases associated with both adverse reactions and ineffectiveness	13 (32.5%)

MDD: major depressive disorder; PTSD: Post-traumatic stress disorder, GAD: general anxiety disorder.

Regarding the antidepressant treatments at the time of the PGx testing request, SSRIs were the most common, accounting for 45.0% (n = 18) of prescriptions, followed by SNRIs at 27.5% (n = 11), atypical antidepressants at 20.0% (n = 8), and TCAs at 17.5% (n = 7). Among the patients, 58.0% (n = 23) were prescribed multiple medications. Specifically, 10.0% (n = 4) received two antidepressants, 50.0% (n = 20) were prescribed an antipsychotic, and 10.0% (n = 4) were given an antiepileptic medication.

The comprehensive analysis of patient prescriptions identified no enzyme inducers or inhibitors, except in the case of psychotropic agents in 20.0% (n = 8) of the patients: fluoxetine (n = 3) and paroxetine (n = 5).

### 3.2 Predicted phenotypes

In our cohort of 40 patients, PGx phenotyping revealed diverse metabolizer statuses for the CYP2D6 and CYP2C19 enzymes. Specifically, 47.5% of patients were identified as NMs for CYP2D6, 40.0%as IMs, 5.0% as PMs, and 7.5% as UMs. For CYP2C19, 35.0% were categorized as NMs, 32.5% as IMs, 2.5% as PMs, 25.0% asRMs, and 5.0% as UMs. Notably, 17.5% of the patients (7 out of 40) exhibited NM phenotypes for both CYP2D6 and CYP2C19.

Regarding treatment suitability based on these phenotypes, 27.5% of patients were receiving treatments that were not recommended according to our phenotyping algorithm. This included 20.0% of patients on treatments with formal contraindications and 7.5% with whom the treatment should be used with caution.

Distribution of CYP2D6 and CYP2C19 phenotypes and participant characteristics, genotype, and predicted phenotype are systematically presented in Tables 2 and 3, respectively.

**TABLE 2:** Distribution of CYP2D6 and CYP2C19 phenotypes for 40 study participants, segmented by ethnicity.

Gene	PM	IM	NM	UM
CYP2D6	Total = 2 (5.0%)	Total = 16 (40.0%)	Total = 19 (47.5%)	Total = 3 (7.5%)
European	2 (5.0%)	15 (37.5%)	14 (35.0%)	2 (5.0%)
Sub-Saharian African		1 (2.5%)	1 (2.5%)	
Oceanian			2 (5.0%)	
East Asian			2 (5.0%)	
Other^a^				1 (2.5%)

	PM	IM	NM	RM	UM
CYP2C19	Total = 1 (2.5%)	Total = 13 (32.5%)	Total = 14 (35.0%)	Total = 10 (25.0%)	Total = 2 (5.0%)
European		10 (25.0%)	11 (27.5%)	10 (25.0%)	2 (5.0%)
Sub-Saharian African			3 (7.5%)		
Oceanian		2 (5.0%)			
East Asian		1 (2.5%)			
Other^a^	1 (2.5%)				

NM: normal metabolizer; IM: intermediate metabolizer; PM: poor metabolizer; RM: rapid metabolizer; UM: Ultra-rapid metabolizer

^a^ Admixtured patient with European and East Asian parents.

**TABLE 3:** Participant characteristics, genotype, and predicted phenotype.

Case	Ethnicity/Ancestry	Sex	Primary diagnosis	Antidepressants	Coprescriptions	Types of response	CYP2D6	CYP2C19
P01	E	M	PTSD	Mirtazapine	Propericiazine	IE	*1/*4 (IM)	*1/*2 (IM)
P02	P	M	PTSD	Duloxetine	Risperidone	IE	*1/*1 (NM)	*1/*2 (IM)
P03	E	F	MDD	Venlafaxine	Quetiapine	ADRs, IE	*2/*4 (IM)	*1/*2 (IM)
P04	E	M	MDD	Escitalopram, Mirtazapine		IE	*1/*9 (NM)	*1/*2 (IM)
P05	As	M	PTSD	Venlafaxine, Mirtazapine	Haloperidol	IE	*1/*10 (NM)	*1/*2 (IM)
P06	E	F	MDD	Escitalopram		ADRs, IE	*5/*9 (IM)	*1/*1 (NM)
P07	E	F	MDD	Paroxetine	Risperidone	ADRs, IE	*1/*1 (NM)	*1/*1 (NM)
P08	E	F	MDD	Venlafaxine		IE	*1/*9 (NM)	*1/*17 (RM)
P09	E	F	MDD	Escitalopram		ADRs, IE	*4/*41 (IM)	*1/*1 (NM)
P10	E	M	MDD	Venlafaxine, Mirtazapine	Risperidone	IE	*4/*4 (PM)	*1/*2 (IM)
P11	E	M	MDD	Fluoxetine	Valproate	IE	*1/*8 (IM)	*2/*17 (IM)
P12	E	M	MDD	Amitryptiline	Aripiprazole	ADRs, IE	*4/*35 (IM)	*1/*2 (IM)
P13	E	M	MDD	Clomipramine	Risperidone	IE	*4/*41 (IM)	*1/*2 (IM)
P14	E	M	MDD	Fluoxetine	Amisulpride	IE	*4/*41 (IM)	*1/*17 (RM)
P15	E	M	MDD	Escitalopram	Lamotrigine	ADRs, IE	*1/*9 (NM)	*17/*17 (UM)
P16	E	F	MDD	Paroxetine		ADRs	*1/*4 (IM)	*1/*1 (NM)
P17	Af	M	PTSD	Clomipramine	Quetiapine, Valpromide	IE	*1/*17 (NM)	*1/*1 (NM)
P18	Af	F	MDD	Venlafaxine		IE	*1/*5 (IM)	*1/*1 (NM)
P19	E	F	MDD	Vortioxetine	Aripiprazole	IE	*1/*4 (IM)	*17/*17 (UM)
P20	E	M	PTSD	Amitryptiline		ADRs, IE	*1/*4 (IM)	*1/*17 (RM)
P21	E	M	PTSD	Paroxetine		ADRs, IE	*2/*41 (NM)	*1/*17 (RM)
P22	E	F	GAD	Venlafaxine	Risperidone	IE	*1/*41 (NM)	*1/*17 (RM)
P23	E	F	MDD	Paroxetine		ADRs, IE	*1/*1 (NM)	*1/*1 (NM)
P24	E	F	MDD	Fluoxetine		ADRs, IE	*1/*1 (NM)	*1/*1 (NM)
P25	E	M	PTSD	Escitalopram, Amitryptiline		IE	*2/*39 (NM)	*1/*17 (RM)
P26	E	M	GAD	Paroxetine		ADRs, IE	*1/*1 (NM)	*1/*1 (NM)
P27	E	M	MDD	Venlafaxine	Risperidone	IE	*1/*4 (IM)	*1/*1 (NM)
P28	P	M	PTSD	Escitalopram		IE	*1/*1 (NM)	*1/*2 (IM)
P29	E	F	MDD	Venlafaxine	Haloperidol	IE	*1/*2 (NM)	*1/*1 (NM)
P30	E	F	Bipolar disorder	Sertraline	Quetiapine, Lamotrigine	IE	*1/*1 (NM)	*1/*1 (NM)
P31	E	M	MDD	Mianserin	Haloperidol	IE	*4/*35 (IM)	*1/*17 (RM)
P32	E	M	GAD	Amitryptiline		IE	*1 × 2/*35 (UM)	*1/*17 (RM)
P33	Af	M	PTSD	Venlafaxine	Risperidone	IE	*1/*1 (NM)	*1/*1 (NM)
P34	E	M	MDD	Escitalopram		ADRs, IE	*1/*4 (IM)	*1/*2 (IM)
P35	E	M	MDD	Mirtazapine		IE	*3/*4 (PM)	*1/*17 (RM)
P36	E	M	MDD	Clomipramine		IE	*2/*39 (NM)	*1/*2 (IM)
P37	E	M	PTSD	Vortioxetine	Aripiprazole	IE	*2/*39 (NM)	*1/*2 (IM)
P38	E	M	PTSD	Venlafaxine	Risperidone	IE	*2/*4 (IM)	*1/*1 (NM)
P39	E	F	MDD	Sertraline		IE	*2 × 2/*1N (UM)	*1/*17 (RM)
P40	Ea	M	PTSD	Sertraline	Quetiapine	ADRs, IE	*2 × 2/*35 (UM)	*2/*3 (PM)

E: european; Af: African; P: polynesian; As: Asian; Ea: Eurasian. NM: normal metabolizer; IM: intermediate metabolizer; PM: poor metabolizer; RM: rapid metabolizer; UM: Ultra-rapid metabolizer; MDD: major depressive disorder; GAD: general anxiety disorder; PTSD: Post-traumatic stress disorder; ADRs: Adverse drug reactions; IE: ineffectiveness.

### 3.3 Case analysis: pairs

#### 3.3.1 Antidepressant-response pairs

SSRIs accounted for 78.6% of ADRs followed by TCAs (14.3%) and SNRIs (7.1%). Regarding cases of ineffectiveness, SSRIs accounted for 39.5%, SNRIs 25.6%, atypical antidepressants 18.6%, and TCAs 15.3% respectively.

In total, SSRIs were responsible for 49.1% of inadequate medication responses, SNRIs 21.1%, TCAs 15.8%, and atypical antidepressants 14.0%. These results are presented in Table 4.

**TABLE 4:** Antidepressant-response pairs.

	ADRs (n = 14)	IE (n = 43)	Total (n = 57)
**Tricyclic antidepressants**	**2 (14.3%)**	**7 (16.3%)**	**9 (15.8%)**
Amitriptyline	2 (14.3%)	4 (9.3%)	6 (10.5%)
Clomipramine	0 (0.0%)	3 (7.0%)	3 (5.3%)
**Selective serotonin reuptake inhibitors**	**11 (78.6%)**	**17 (39.5%)**	**28 (49.1%)**
Escitalopram	4 (28.6%)	7 (16.3%)	11 (19.3%)
Fluoxetine	1 (7.1%)	3 (7.0%)	4 (7.0%)
Paroxetine	5 (35.7%)	4 (9.3%)	9 (15.8%)
Sertraline	1 (7.1%)	3 (7.0%)	4 (7.0%)
**Selective norepinephrine reuptake inhibitors**	**1 (7.1%)**	**11 (25.6%)**	**12 (21.1%)**
Duloxetine	0 (0.0%)	1 (2.3%)	1 (1.8%)
Venlafaxine	1 (7.1%)	10 (23.3%)	11 (19.3%)
**Atypical antidepressants**	**0 (0.0%)**	**8 (18.6%)**	**8 (14.0%)**
Mianserin	0 (0.0%)	1 (2.3%)	1 (1.8%)
Mirtazapine	0 (0.0%)	5 (11.6%)	5 (8.8%)
Vortioxetine	0 (0.0%)	2 (4.7%)	2 (3.5%)

ADRs: Adverse drug reactions; IE: ineffectiveness.

#### 3.3.2 Gene-drug/antidepressant-responses pairs

Out of the 40 cases examined, eleven were not included in this analysis due to the antidepressants they were prescribed not being reviewed by CPIC or being assigned a lower level of evidence (despite the potential implications of CYP2D6 and CYP2C19 in their metabolism pathway). Specifically, these antidepressants included venlafaxine (which only has recommandations for CYP2D6 PM but not for UM or IM phenotypes), duloxetine, fluoxetine, mirtazapine, and mianserin (CPIC, 2023; Bousman et al., 2023). The cases excluded for such reasons were P01, P02, P03, P11, P14, P18, P24, P27, P31, P35 and P38.

Of the remaining 29 cases, 12 (41.4%) were CYP2D6 non-NMs and 19 (65.6%) were CYP2C19 non-NMs. A total of 50 gene-drug-response pairs were identified involving the antidepressant drug classes SSRIs, TCAs, and SNRIs. The specific drugs included amitriptyline, clomipramine, paroxetine, sertraline, escitalopram, venlafaxine and vortioxetine (Table 5).

**TABLE 5:** Actionable CYP2D6/CYP2C19-antidepressant-response pairs.

	Actionable pairs (n = 50)
Drug class (CPIC evidence level)	
All TCAs	
CYP2D6-Amitriptyline (A)	3 (50.0%)
CYP2C19-Amitriptyline (A)	4 (66.7%)
CYP2D6-Clomipramine (B)	0 (0.0%)
CYP2C19-Clomipramine (B)	0 (0.0%)
All SSRIs	
CYP2D6-Paroxetine (A)	0 (0.0%)
CYP2C19-Sertraline (B)	1 (25.0%)
CYP2C19-Escitalopram (A)	3 (27.3%)
SNRIs	
CYP2D6-Venlafaxine (A/B)	0 (0.0%)
Atypical	
CYP2D6-Vortioxetine (A/B)	0 (0.0%)
Pharmacogene	
CYP2D6	3 (11.5%)
CYP2C19	8 (33.3%)
Drug response phenotype	
ADRs	5 (35.7%)
IE	6 (16.7%)

TCAs: Tricyclic antidepressants; SSRIs: Selective serotonin reuptake inhibitors; SNRIs: Selective norepinephrine reuptake inhibitors; ADRs: Adverse drug reactions; IE: ineffectiveness.

Among the 50 CYP2D6/CYP2C19-antidepressant-response pairs analyzed, the PGx of CYP2D6 and CYP2C19 were potentially explanatory for a total of 11 pairs (22.0%), rendering them actionable. The actionability of CYP2D6 was 3/26 = 11.5% and 8/24 = 33.3% for CYP2C19. These results are presented in Supplementary Table S1.

Across various classes of antidepressant drugs, the proportion of actionable pairs was variable: none for atypical antidepressants and SNRIs, 38.9% (n = 7) for TCAs, and 16.7% (n = 4) for SSRIs.

Out of the 14 observed CYP2D6/CYP2C19-antidepressant-ADR pairs, 35.7% (n = 5) were actionable, whereas the proportion for pairs showing ineffectiveness was lower at 16.7% (n = 6).

Table 5 summarizes the actionability of CYP2D6/CYP2C19-antidepressant-response pairs with respect to CPIC evidence level.

### 3.4 Utilization of algorithm recommendations by psychiatrists

#### 3.4.1 PGx algorithm recommendations

The algorithm recommendations were followed by psychiatrists in 92.5% (n = 37) of cases. Even in cases where the pairs were not actionable, practitioners used the algorithm recommendations to adjust the prescription based on the predicted phenotypes and recommended treatments. It should be noted that these recommendations, given at the time of PGx prescriptions (i.e., between June 2020 and April 2022), were based on previous CPIC guidelines (Baldacci et al., 2022).

In practice, we observed that the prescribed treatment was continued in 42.5% (n = 17) of cases, with a dose increase in 29.4% (n = 5) of patients. Another molecule was added in 17.5% (n = 7) of cases. Treatment was switched in 40.0% (n = 16) of cases. The changes or additions involved SSRIs in 56.5% (n = 13) of cases, mianserin or mirtazapine in 13.0% (n = 3), an antiepileptic in 8.7% (n = 2), an antipsychotic in 8.7% (n = 2), or an SNRI in 4.3% (n = 1).

According to the practitioners, 71.4% (n = 10) of patients showed a significant diminution (≥3/5) in ADRs. For efficacy, psychiatrists reported improvement (≥3/5) in 79.5% (n = 31) of patients. These results are presented as (Supplementary Table S2).

All the psychiatrists reported believing that PGx testing helped them in their practice and were willing to use it in the future.

#### 3.4.2 Illustrative case reports

The following are two case examples from our cohort, selected to illustrate the use of PGx testing in addressing antidepressant ineffectiveness and adverse drug reactions.

A 55-year-old European man with PTSD (P25) was prescribed escitalopram and amitriptyline. Due to ineffectiveness, benefited from PGx testing. The predicted phenotype for CYP2D6 and CYP2C19 was NM (*2/*39) and RM (*1/*17), respectively. According to our analysis, he presented the following two actionable pairs: CYP2C19-amitriptyline-ineffectiveness and CYP2C19-escitalopram-ineffectiveness. Following the recommendations of the PGx algorithm, the practitioner changed the previous treatment to paroxetine. He reported a moderate improvement, rated as 3/5 on the visual analogue scale.

A second patient (P40) was a 39-year-old European male with PTSD who was prescribed sertraline and quetiapine. Due to ineffectiveness and ADRs (weight gain, somnolence, asthenia), he underwent the PGx testing. The predicted phenotype for CYP2D6 and CYP2C19 was UM (*2 × 2/*35) and PM (*2/*3), respectively. Our case analysis revealed one actionable pair: CYP2C19-sertraline-ADRs. Following the recommendations of the PGx algorithm, the practitioner replaced sertraline with mirtazapine. According to his report, the subject experienced a very mild improvement in ADRs, rated as 1/5, and a mild improvement in efficacy, rated as 2/5 on the visual analogue scales.

## 4 Discussion

This case series study describes a population of 40 patients who underwent PGx testing due to ineffectiveness or ADRs associated with antidepressant treatment. One of the strengths of this study is the comprehensive database, encompassing both clinical and genotypic information. It is noteworthy that 97.5% of the cumulative requests were related to ineffectiveness and 35.0% to ADRs, with 32.5% of requests being for both indications simultaneously. These figures differ from those of Kee et al. (2023), who found in their study on PGx testing, 40% of indications for ineffectiveness and 79% for ADRs, with an overlap of both indications in 19% of cases. These differences could be explained by various aspects related to the study population and conditions: Kee et al. (2023) took cases from the Understanding Adverse Drug Reactions Using Genomic Sequencing (UDRUGS) study that focused on ADRs, which likely influenced patient recruitment (Maggo et al., 2019; Kee et al., 2023). Our cohort in that recruitment was open: psychiatrists referred patients for ineffectiveness, ADRs, or both, making our population likely to be more representative of the population who could benefit from PGx testing in a real-life psychiatric setting. As our population consisted mostly (90.0%) of MDD and PTSD, conditions particularly resistant to treatments (Rush et al., 2006; Hoskins et al., 2015), it is hardly surprising that the indication of ineffectiveness predominates.

Moreover, we logically observe that in this population, predominantly composed of patients with treatment-resistant psychiatric disorders, 50% also have a prescription for antipsychotics. As the indications overlap and many real-life patients receive co-prescriptions of different classes of psychotropic medications, this demonstrates a need to develop PGx algorithms that incorporate both antipsychotics and antidepressants, as our team has done (Baldacci et al., 2023). Similarly, considering that 27.5% of patients were receiving a treatment that was not compatible with the predicted phenotype, the implementation of PGx testing at the initial prescription stage (i.e., before selecting the medication) clearly offers potential benefits for the psychiatric patient population.

The genotyping for *CYP2D6* and *CYP2C19* revealed an increased frequency of certain phenotypic profiles. According to PharmGKB, the proportions in the general European population are approximately 2.5% CYP2D6 UMs, 51.0% CYP2D6 NMs, 38.9% CYP2D6 IMs, and 6.5% CYP2D6 PMs (Whirl-Carrillo et al., 2012; PharmGKB, 2017). While our results were quite similar for CYP2D6 NMs (47.5%), CYP2D6 IMs (40.0%), and CYP2D6 PMs (5.0%), the proportion of CYP2D6 UMs appeared considerably greater (7.5%, approximately three times higher than in the general population). This finding is noteworthy because these three CYP2D6 UM patients were included due to lack of therapeutic efficacy, as observed in other studies, in which this metabolic profile has been shown to be associated with treatment ineffectiveness (Ingelman-Sundberg, 2005). Given the limited size of our study, further research must be conducted to confirm the overrepresentation of UMs in the population of antidepressant-resistant patients. Similarly, according to PharmGKB, the proportions in the general European population are approximately 4.7% CYP2C19 UMs, 27.2% CYP2C19 RMs, 39.6% CYP2C19 NMs, 26.2% CYP2C19 IMs, and 2.4% CYP2C19 PMs (Whirl-Carrillo et al., 2012; PharmGKB, 2015). Our results are similar for CYP2C19 UMs (5.0%), CYPC19 RMs (25.0%), NMs (35.0%), CYP2C19 IMs (32.5%), and CYP2C19 PMs (2.5%). Finally, contrary to our hypothesis, apart from the overrepresentation of CYP2D6 UMs, which may be due to the small sample size, the population in our sample, made up of patients receiving antidepressant treatment in our psychiatric department, was not significantly different from the general European population for the CYP2D6 and CYP2C19 phenotypes.

Another way to consider these results is to examine the prevalence of NM patients in our sample. In the 40 cases included for CYP2D6/CYP2C19-antidepressant-response pair analysis, 52.5% exhibited CYP2D6 non-NMs and 65.0% had CYP2C19 non-NMs. These findings are consistent with previous studies by Maggo et al. (2019), who reported similarly high percentages of non-NMs for CYP2D6 (42.7%) and CYP2C19 (64%) (Maggo et al., 2019) and with Kee et al. (2023) who found that 57% were CYP2D6 non-NMs and 62% were CYP2C19 non-NMs in their cohort. In a large Danish population-based case cohort study of patients with mental disorders, including depression (n = 51,464), it was found that 73% of the cases exhibited CYP2D6 and CYP2C19 non-NM phenotypes (Lunenburg et al., 2021). However, in their research, Kee et al. (2023) found that only 15% of NM phenotypes were present in both CYP2D6 and CYP2C19, which closely aligns with our findings. We observed that approximately 17.5% of patients treated with an antidepressant in our psychiatry department possess NM phenotypes in both CYP2D6 and CYP2C19. These findings confirm the importance of integrating PGx testing into the process of selecting antidepressant therapy, especially during the initial prescription, to mitigate the risk of ineffectiveness or ADRs within a patient population where the majority are non-NM.

We identified that 22.0% of the CYP2D6/CYP2C19-antidepressant-response pairs were actionable, with 11.5% attributed to CYP2D6 and 33.3% to CYP2C19. This contrasts with the findings of Kee et al. (2023), who reported 38% of actionable pairs. Our results are closer to those obtained by Hahn and Roll (2023), who reported proportions of CYP2D6 and CYP2C19 actionable genotypes of 17% and 37%, respectively. However, it should be noted that in our study, we relied on the 2023 CPIC recommendations (Bousman et al., 2023; Baldacci et al., 2023), while Kee et al. (2023) based their study on recommendations from 2019 (Bousman et al., 2019) or even, on more isolated scientific data when CPIC did not make a recommendation, on. This approach may lead to variations in the results, as the CPIC update in 2023 does not provide exactly the same recommendations as in the previous version. For example, in 2023, venlafaxine has dose adjustment recommendations for individuals with a PM phenotype for CYP2D6, but there are no recommendations for UM or IM phenotypes. Additionally, recommendations for sertraline and paroxetine have been modified (IM for CYP2D6-paroxetine-ADR and RM for CYP219-sertraline-IE are now considered “non-actionable”), which differs from the approach followed by Kee et al. (2023). If we had used exactly the same methodology as Kee et al. (2023), we would have found that 27.3% of the CYP2D6/CYP2C19-antidepressant-response pairs were actionable, with 19.4% attributed to CYP2D6 and 37.5% to CYP2C19. This underscores the importance of the medical community relying solely on recommendations with high levels of evidence from international expert societies. In this regard, our definition of actionability aligns more closely with that used in the study by Hahn and Roll (2023), which is based solely on the most authoritative recommendations.

In accordance with our hypotheses, results from this real-world case series demonstrate the predictive significance of CYP2D6 and CYP2C19 PGx. One surprising result is that despite the initial difference in our database compared to Kee et al. (2023) in terms of the number of pairs, we find very similar ratio when considering ADRs and ineffectiveness independently. Thus, among the CYP2D6/CYP2C19-antidepressant-ADRs pairs observed, 35.7% were actionable in our cohort (and 48% for Kee et al.), while the proportion for ineffectiveness pairs was 16.7% (21% for Kee et al.) This result is of particular interest insofar as it seems to confirm that, despite the difference in population, PGx testing explains ADRs and ineffectiveness in the same proportions for the psychiatric patient population. If the proportion of ADRs is typically twice that of ineffectiveness as explained by PGx, this could be attributed to their direct association with medication intake and its metabolic tolerance, which are heavily influenced by genetics. Conversely, the ineffectiveness of antidepressants may be influenced by various factors, such as illness severity, individual patient traits, environmental elements, and treatment adherence. While PGx may also contribute to antidepressant efficacy, it is generally believed to have a greater impact on the occurrence of ADRs due to its direct correlation with drug metabolism and individual response to medication. This is supported by existing literature, where the genotypes of both CYP2D6 and CYP2C19 have been extensively linked to the tolerability profile of antidepressants (Schenk et al., 2010; Huezo-Diaz et al., 2012; Chang et al., 2014; Milosavljevic et al., 2021; Jukić et al., 2018; Han et al., 2018; Kee et al., 2023; Bousman et al., 2019; Lunenburg et al., 2021).

The use of a PGx algorithm is easy to implement within a Psychiatry department with a laboratory (Baldacci et al., 2022; Baldacci et al., 2023). In routine practice, PGx datas complete the clinical features to guide the prescription. The final decision rests with the psychiatrist. If the current treatment is not recommended by the algorithm, it is classicaly swiched for a recommanded one. In the case where the current treatment is recommanded, a dose adjustment or a switch is possible.

The two illustrative cases demonstrate how PGx guidance could offer valuable alternatives, providing practitioners with data-driven recommendations to optimize treatment and enhance patient outcomes. PGx testing not only identified the genetic factors influencing drug responses but also supported practitioners in making more informed, personalized decisions for each patient. Our study demonstrates that psychiatrists’ adherence to this type of prescription guideline is very important (the recommendations were followed in 92.5% of cases, even when the current prescription was not actionable) and this attests to the strong interest in personalized medicine among physicians. According to some authors, the impact of PGx appears to be greater (larger effect size) in reducing side effects than in improving treatment efficacy (Pérez et al., 2017; Han et al., 2018). In our study, we did not observe any noteworthy difference in the assessment of ADRs improvement compared to efficacy evaluation (the improvement rates were 71.4% and 79.5% for the respective patient groups).

The limitations of this study can be categorized into four main aspects. Firstly, we focused on genotyping common variants, for *CYP2D6* and *CYP2C19*, known to be associated with variable drug pharmacokinetics. It is plausible that some patients may carry novel variants within the genes taken into consideration or other genes, impacting their response to prescribed medication. Secondly, our case cohort is relatively small (n = 40), and we opted not to exclude the 5 patients of non-European descent; consequently, we did not stratify variant analyses by population ancestry. This decision was made to reflect the phenotypic diversity of patients received in routine practice at a European hospital. Thirdly, as highlighted by Kee et al. (2023), the extent of association between genotype-inferred phenotypes, drug exposure, and drug response events utilized in establishing the actionability for gene-antidepressant-response pairs remains uncertain. Even though we followed the most recent scientific recommendations from the CPIC (Bousman et al., 2023), the observed response event might be influenced in part by other factors that were unknown at the time of recruitment, potentially impacting the association. Fourthly, the assessment of improvement is prone to bias. Improvement was subjectively rated by the practitioner, in the absence of an objective scale, with an evident bias as shown by the high adherence rate. Furthermore, depending on the therapeutic relationship, the explanation provided to the patient when choosing treatment supported by objective PGx data may also contribute to enhancing the placebo effect and patient adherence to their treatment. Clinical improvement is obviously multifactorial, and it is difficult to attribute it solely to drug prescription. Nevertheless, PGx testing provides a means to explain the ineffectiveness and ADRs related to certain antidepressant treatments. The genetic report was also relevant as a basis for dosage adjustments in order to mitigate ADRs. The prescribers adhered to the recommendations provided in the PGx reports and found value in this tool, as evidenced by the high rate of compliance. PGx testing could find a place in an approach combined with an algorithm enhanced with certain clinical and pharmacological data, to help guide prescriptions.

In conclusion, our findings suggest that PGx testing and referral of mental health patients experiencing ineffectiveness or ADRs can yield a high rate of gene variants potentially explaining these poor treatment outcomes. These observations highlight the potential value of providing PGx data for such selected patients. However, further large-scale studies are warranted to comprehensively assess the impact on treatment outcomes, evaluate the cost-effectiveness of this approach as well as its relevance in the context of initial antidepressant prescription.

## Data Availability

The original contributions presented in the study are included in the article/Supplementary Material, further inquiries can be directed to the corresponding author.
