# Supplementary material for: Pharmacogenetics testing for poor response to antidepressants: a transnosographic case series
Source: Front Pharmacol. 2024 Oct 9;15:1440523. doi: 10.3389/fphar.2024.1440523 (PMC11496244; doi:10.3389/fphar.2024.1440523)
Supplement: Supplementary file 1 [file Table1.DOCX]

***Supplementary Material***

Pharmacogenetics testing for poor response to antidepressants: a transnosographic case series

**Marie-Agnès Lorvellec^1^** †, **Gilles Sipahimalani^1^** †**, Bertrand Lahutte^1,2^, Hervé Delacour^2,3^, Antoine Baldacci^1^, Emeric Saguin^1,4^**

^1^ Department of Psychiatry, Bégin Military Teaching Hospital, 94160 Saint-Mandé, France

^2^ Ecole du Val-de-Grâce, French Military Medical Academy, Paris, France

^3^ Biological Unit, Bégin Military Teaching Hospital, 94160 Saint-Mandé, France

^4^ UMR 7330 VIFASOM, Hôtel-Dieu, APHP, Paris, France

†These authors contributed equally to this work and share first authorship

*** Correspondence:**Emeric SAGUIN
[saguinemeric@gmail.com](mailto:saguinemeric@gmail.com)

| **Table S1: CYP2D6/CYP2C19-antidepressant-response pairs**  NM: normal metabolizer; IM: intermediate metabolizer; PM: poor metabolizer; RM: rapid metabolizer; UM: ultra-rapid metabolizer; ADRs: Adverse drug reactions; IE: ineffectiveness | | | | | |
| --- | --- | --- | --- | --- | --- |
| **No** | **Case** | **CYP2D6** | **CYP2C19** | **Gene-antidepressant-response pairs** | **Actionability** |
| 1 | P04 | Normal | Intermediate | CYP2C19-Escitalopram-IE |  |
| 2 | P05 | Normal | Intermediate | CYP2D6-Venlafaxine-IE |  |
| 3 | P06 | Intermediate | Normal | CYP2C19-Escitalopram-ADR |  |
| 4 |  |  |  | CYP2C19-Escitalopram-IE |  |
| 5 | P07 | Normal | Normal | CYP2D6-Paroxetine-ADR |  |
| 6 |  |  |  | CYP2D6-Paroxetine-IE |  |
| 7 | P08 | Normal | Rapid | CYP2D6-Venlafaxine-IE |  |
| 8 | P09 | Intermediate | Normal | CYP2C19-Escitalopram-ADR |  |
| 9 |  |  |  | CYP2C19-Escitalopram-IE |  |
| 10 | P10 | Poor | Intermediate | CYP2D6-Venlafaxine-IE |  |
| 11 | P12 | Intermediate | Intermediate | CYP2D6-Amitryptiline-ADR | √ |
| 12 |  |  |  | CYP2D6-Amitryptiline-IE |  |
| 13 |  |  |  | CYP2C19-Amitryptiline-ADR | √ |
| 14 |  |  |  | CYP2C19-Amitryptiline-IE |  |
| 15 | P13 | Intermediate | Intermediate | CYP2D6-Clomipramine-IE |  |
| 16 |  |  |  | CYP2C19-Clomipramine-IE |  |
| 17 | P15 | Normal | Ultrarapid | CYP2C19-Escitalopram-ADR |  |
| 18 |  |  |  | CYP2C19-Escitalopram-IE | √ |
| 19 | P16 | Intermediate | Normal | CYP2D6-Paroxetine-ADR |  |
| 20 | P17 | Normal | Normal | CYP2D6-Clomipramine-IE |  |
| 21 |  |  |  | CYP2C19-Clomipramine-IE |  |
| 22 | P19 | Intermediate | Ultrarapid | CYP2D6-Vortioxetine-IE |  |
| 23 | P20 | Intermediate | Rapid | CYP2D6-Amitryptiline-ADR | √ |
| 24 |  |  |  | CYP2D6-Amitryptiline-IE |  |
| 25 |  |  |  | CYP2C19-Amitryptiline-ADR |  |
| 26 |  |  |  | CYP2C19-Amitryptiline-IE | √ |
| 27 | P21 | Normal | Rapid | CYP2D6-Paroxetine-ADR |  |
| 28 |  |  |  | CYP2D6-Paroxetine-IE |  |
| 29 | P22 | Normal | Rapid | CYP2D6-Venlafaxine-IE |  |
| 30 | P23 | Normal | Normal | CYP2D6-Paroxetine-ADR |  |
| 31 |  |  |  | CYP2D6-Paroxetine-IE |  |
| 32 | P25 | Normal | Rapid | CYP2D6-Amitryptiline-IE |  |
| 33 |  |  |  | CYP2C19-Amitryptiline-IE | √ |
| 34 |  |  |  | CYP2C19-Escitalopram-IE | √ |
| 35 | P26 | Normal | Normal | CYP2D6-Paroxetine-ADR |  |
| 36 |  |  |  | CYP2D6-Paroxetine-IE |  |
| 37 | P28 | Normal | Intermediate | CYP2C19-Escitalopram-IE |  |
| 38 | P29 | Normal | Normal | CYP2D6-Venlafaxine-IE |  |
| 39 | P30 | Normal | Normal | CYP2C19-Sertraline-IE |  |
| 40 | P32 | Ultrarapid | Rapid | CYP2D6-Amitryptiline-IE | √ |
| 41 |  |  |  | CYP2C19-Amitryptiline-IE | √ |
| 42 | P33 | Normal | Normal | CYP2D6-Venlafaxine-IE |  |
| 43 | P34 | Intermediate | Intermediate | CYP2C19-Escitalopram-ADR | √ |
| 44 |  |  |  | CYP2C19-Escitalopram-IE |  |
| 45 | P36 | Normal | Intermediate | CYP2D6-Clomipramine-IE |  |
| 46 |  |  |  | CYP2C19-Clomipramine-IE |  |
| 47 | P37 | Normal | Intermediate | CYP2D6-Vortioxetine-IE |  |
| 48 | P39 | Ultrarapid | Rapid | CYP2C19-Sertraline-IE |  |
| 49 | P40 | Ultrarapid | Poor | CYP2C19-Sertraline-IE |  |
| 50 |  |  |  | CYP2C19-Sertraline-ADR | √ |
| NM: normal metabolizer; IM: intermediate metabolizer; PM: poor metabolizer; RM: rapid metabolizer; UM: ultra-rapid metabolizer | | | | | |

| **Table S2. Clinical decisions and outcomes** | | | | |
| --- | --- | --- | --- | --- |
|  | **Total** | **IE (n=26)** | **ADRs (n=1)** | **IE + ADRs (n=13)** |
| **Recommandations followed** | 37 (92.5%) | 25 (96.1%) | 1 (100.0%) | 11 (84.6%) |
| **Treatment persisted** | 17 (42.5%) | 13 (50.0%) | 1 (100.0%) | 3(23.1%) |
| Dosage increase | 5 (29.4%) | 4 (30.8%) | 0 (0.0%) | 1 (33.3%) |
| **Treatment addition** | 7 (17.5%) | 6 (23.1%) | 0 (0.0%) | 1 (7.7% ) |
| **Switch** | 16 (40.0%) | 7 (26.9%) | 0 (0.0%) | 9 (69.2%) |
| Added or switched treatment |  |  |  |  |
| SSRIs | 13 (56.5%) | 8 (61.5%) | 0 (0.0%) | 5 (50.0%) |
| SNRIs | 1 (4.3%) | 0 (0.0%) | 0 (0.0%) | 1 (10.0%) |
| Atypical antidepressants | 3 (13.0%) | 2 (15.4%) | 0 (0.0%) | 1 (10.0%) |
| Antipsychotics | 2 (8.7%) | 0 (0.0%) | 0 (0.0%) | 2 (20.0%) |
| Antiepileptics | 2 (8.7%) | 2 (15.4%) | 0 (0.0%) | 0 (0.0%) |
| **Clinical improvement** | 31 (79.5%) | 20 (76.9%) |  | 11 (84.6%) |
| **Reduction in ADRs** | 10 (71.4%) |  | 1 (100.0%) | 9 (69.2%) |
| ADRs: Adverse drug reactions; IE: ineffectiveness; SSRIs: selective serotonin reuptake inhibitors; SNRIs: Selective norepinephrine reuptake inhibitors | | | | |
